# Cell-Extracellular Matrix Feedback Results in Spontaneous Cell Polarization and Heterogeneous Remodeling in 3D Isotropic and Aligned Discrete-Fiber Models of Cell-Mediated Remodeling

**DOI:** 10.1007/s12195-026-00922-0

**Published:** 2026-06-20

**Authors:** Adam W. Y. Ley, Lauren M. Bersie-Larson, Sabin Adhikari, Paolo P. Provenzano, Kevin D. Dorfman, Victor H. Barocas

**Affiliations:** 1 Department of Biomedical Engineering, University of Minnesota – Twin Cities, Minneapolis, MN USA; 2 Department of Chemical Engineering and Materials Science, University of Minnesota – Twin Cities, Minneapolis, MN USA

**Keywords:** Collagen, Biomechanics, Mechanobiology, Pseudopod, Retraction

## Abstract

**Purpose:**

The extracellular matrix (ECM) is a dynamic fiber environment containing structural information that significantly impacts cell behavior. Recent experimental work has demonstrated that cells also have significant capacity to remodel their microenvironment, often resulting in ECM heterogeneity. We present an open-source molecular dynamics platform that simulates cell-mediated remodeling wherein cells plasticly remodel their microenvironment and respond to induced structural heterogeneity over multiple retraction cycles.

**Methods:**

The model applies a coarse-grained discrete fiber approach to cell-mediated remodeling. The ECM, represented by a bead-spring polymer, allows proximity-mediated fiber-fiber interactions, representing fiber crosslinking and entanglement. A simulated cell interprets the heterogeneity of its local microenvironment with variable sensitivity and exhibits anisotropic behavior informed by its microenvironment. The cells generate tractors, representing pseudopods, that bind to the ECM and retract towards the cell surface, causing ECM displacement. The cell detaches from the ECM by deleting tractors and allowing the ECM to relax before re-interpreting its surroundings and repeating this process. Metrics of ECM remodeling (fiber densification, orientation, bond strain) and cell morphology were recorded throughout the simulation. The model was extended to a cell remodeling fiber networks with different levels of pre-existing alignment.

**Results:**

The addition of plasticity in the model enables measurable remodeling: increasing fiber density close to the cell, reorienting fibers radially, and increasing residual fiber bond strain over time. These patterns of remodeling were consistent with previously published experimental results. In initially unaligned fiber networks, cell remodeling resulted in ECM heterogeneity that depended on distance from the cell surface and alignment with the cell’s primary axis. In aligned networks, pre-alignment and sensitivity synergized to increase the heterogeneity of the remodeled networks at further distances from the cell surface.

**Conclusions:**

These findings suggest that cell-ECM feedback mechanisms contribute to heterogeneous remodeling patterns and illustrate that pre-existing alignment impacts remodeling patterns far from the cell. Further, the model presented herein provides a novel modular platform for further investigations into cell-ECM sensing and ECM remodeling heterogeneity.

**Supplementary Information:**

The online version contains supplementary material available at 10.1007/s12195-026-00922-0.

## Introduction

The extracellular matrix (ECM) is a complex microenvironment comprised of fibrous structural proteins like collagen [1]. Early work established that ECM fiber organization induces tissue-scale mechanical anisotropy [2], with higher observed stiffness in the direction of fiber alignment. Aligned structures also have cellular-level impacts, causing cells to preferentially polarize and migrate along pre-existing aligned structures [3, 4]. Such behavior, termed *contact guidance* [5], is posited to arise from local mechanical anisotropy stemming from structural anisotropy. The ability of cells to reorganize the ECM has also been recognized, particularly in seminal *in vitro* work from the 1980s [6–8]. Further work has reinforced this view and increasingly recognized the importance of directionally-mediated cellular processes [9, 10]. Pairs of contractile cells generate highly organized, dense structures [11] of aligned fibers [12], which may enable long-distance force transmission between cells [12, 13]. Similarly, the presence of these aligned structures alter cell behaviors, causing preferential migration along aligned fibers and even increasing cell migration speed [14, 15]. As such, there is a need for models that incorporate the development of aligned structures and the effects they have on cells and the microenvironment.

Early computational models of ECM remodeling used continuum-based approaches to describe feedback between cells and the surrounding ECM. In the 1990s, Barocas and Tranquillo [16] developed an anisotropic biphasic theory that described cell-mediated ECM remodeling via an ECM-informed model of anisotropic traction force generation. Other continuum models continue to be developed to describe the phenomena in applications including angiogenesis [17, 18] and cell migration [19]. Continuum models cannot, however, account for non-affine deformations [20, 21] characteristic of fibrillar networks and do not allow for analysis of cell-scale microstructural changes.

Discrete-fiber computational models of ECM have emerged as a means to understand non-affine fiber network deformations. Many models [22–24] are constrained to two dimensions by computational limitations. Recent three-dimension models have incorporated elements of fiber-fiber interactions [25, 26], such as fiber entanglement and crosslinking. Other models enable interrogation of network topology [27], facilitating quantitative characterization of mechanical and steric ECM anisotropy. There is, however, still need for 3D models that incorporate ECM remodeling *and* interrogation, in which cells generate and respond to dynamic microenvironmental changes. Further, many existing models make use of in-house codes and are not readily adaptable to parallelization or cluster-based computation. To address this need, we developed a 3D discrete-fiber model of cell-mediated ECM compaction that can integrate fiber-fiber binding and cell-ECM interactions in an open-source format that can be readily customized and integrated into multiscale pipelines using cluster-based computing.

## Methods

### Model Development

The model is comprised of three elements: (i) a self-interacting bead-spring representation of ECM; (ii) an ellipsoidal cell that generates contractile elements, called “tractors,” representing pseudopodia; and (iii) specified cell response to ECM alignment. Throughout the simulation, tractors were generated in the ECM far from the cell, bound to fibers, retracted towards the cell via controlled displacement, and finally detached from ECM. Fibers that contacted during the simulation formed new bonds, resulting in plastic changes to ECM topology.

#### Network Generation

ECM networks were generated by constructing a Voronoi or Delaunay tessellation on uniformly random seeded points in a computational volume using Python’s built-in Voronoi and Delaunay tessellation codes. The resulting tessellation edges were then treated as fibers and the intersections as pre-existing crosslinks within the fiber network. The fiber volume fraction φ (Eq. 1) of the network was then determined by

1 $$\upvarphi =\frac{\sum_{f}{l}_{f}\pi {r}^{2}}{V}$$

where f are fibers in the network, l_f_ is the length of each fiber, V is the volume of the simulated space, and r is the radius of the simulated fibers, taken to be 100nm [28]. All networks were generated to have a volume fraction similar to a 1 mg/mL collagen hydrogel [25]. The generation volume typically had 8x the volume of the intended simulation volume to mitigate edge effects.

Alignment in networks can be induced by stretching the network in the coordinate directions. Stretching the network in one direction produces linearly aligned networks while stretching in two directions can create planarly aligned networks. In this study, uniaxial stretch ratios of 1, 1.05, 1.1, 1.25, 1.5, and 2 were explored.

#### ECM parameters and plasticity

Tessellation edges were modeled as cylindrical ECM fibers of 100 nm radius [28], and tessellation nodes formed pre-existing fiber crosslinks. Networks were cropped into 100-μm spheres that placed initially spherical cells 90 μm, or 4.5 cell-lengths, away from the simulation boundaries. Aligned networks were generated similarly but were aligned by stretching in the x-direction by the their stretch ratio before cropping. Delaunay-tessellation-based networks were also generated to explore how topological differences alter ECM remodeling.

**Fig. 1:**
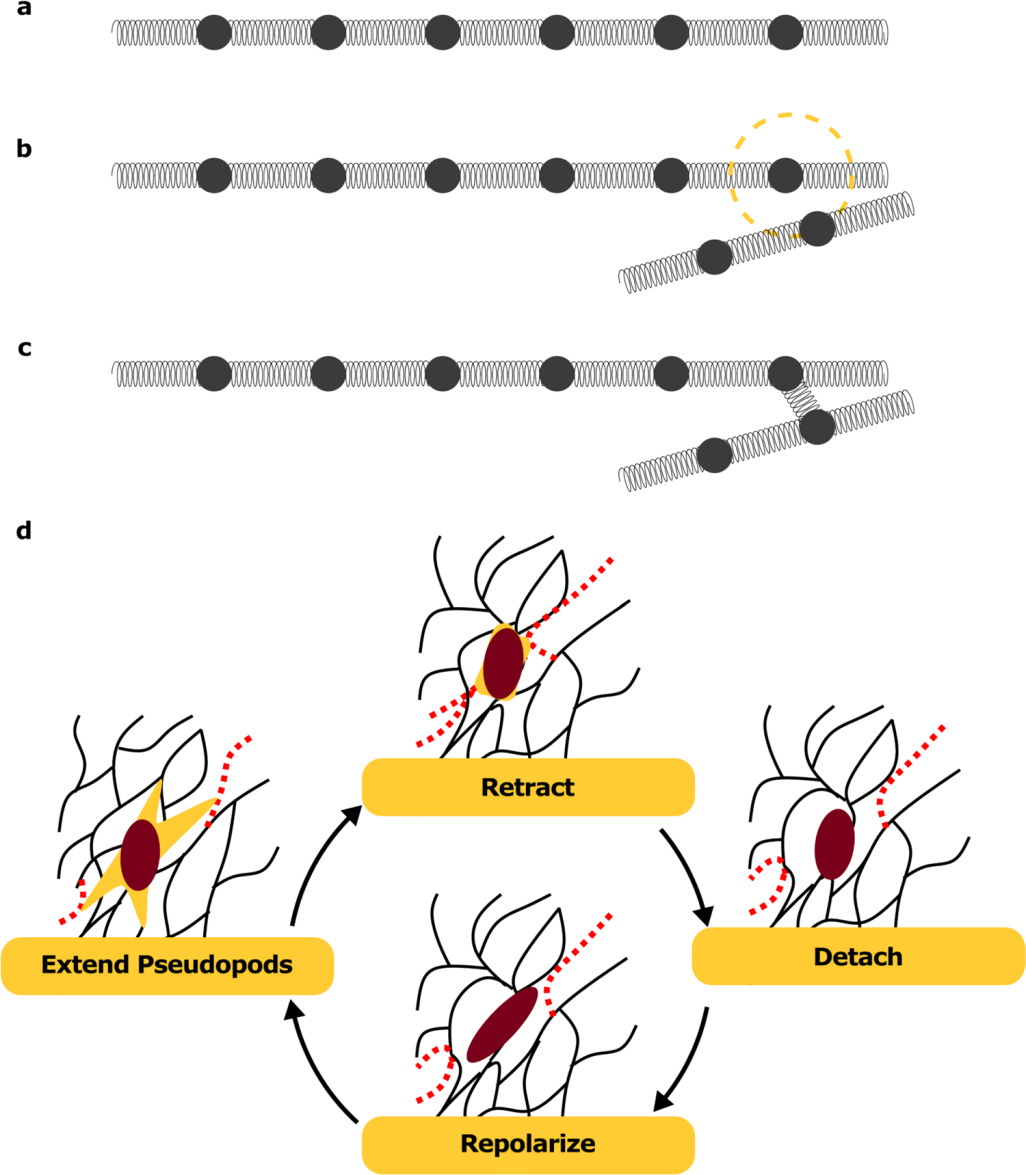
**a** Network edges were segmented into equidistant beads and connected via harmonic springs. **b** Beads from different fibers are prevented from overlapping via Weeks-Chandler-Anderson potentials. **c** In plastic simulations, contacting fibers form new crosslinks. **d** Schematic of cell-driven remodeling.

Each tessellation edge was then segmented into equally spaced segments by dividing the edge length by 400 nm and rounding to the next integer. For instance, a 1700 nm edge would be divided into five equal 340 nm segments. Beads representing ECM fibers were placed along the tessellation edge and connected by harmonic springs (Fig. 1a) (Eq. 2),

2 $${E}_{bond}(r)=K{\left(r-{r}_{0}\right)}^{2}$$

where E_bond_ is the bond energy, K is the spring constant, r is the distance between beads, and r_0_ is the bond rest length, 400 nm. This method put the fibers in the network under initial compression that was later relaxed to form a network that had no initial bond strain. Since the tractors move via controlled displacement, the units of K are arbitrary in nature if the focus is on kinematics and not force generation. Bending of fiber segments was not included in this simulation set; when the fibers were loaded in compression, fiber segments were able to buckle up to the point that two beads came within a fiber diameter (200 nm) of one another but then repulsed according to pair-wise interaction detailed next.

All ECM beads interacted pairwise via a Weeks-Chandler-Anderson (WCA) potential [29] (Fig. 1b) (Eq. 3),

3 $${E}_{pair}(r)=\left\{\begin{array}{ll}4\varepsilon \left[{\left(\frac{\sigma }{r}\right)}^{12}-{\left(\frac{\sigma }{r}\right)}^{6}\right], &\quad r<{r}_{cf}\\ 0, &\quad r\ge {r}_{cf}\end{array}\right.$$

Where ε is a force scaling factor, σ adjusts the length scale of the interaction by shifting the minimal energy distance to $\sqrt[6]{2}*\upsigma$, and *r*_*cf*_ sets the cutoff distance for the interaction corresponding with the 200 nm fiber diameter. This form creates strong repulsive forces between nearby beads, weak attractive interactions at moderate separation, and no interaction between beads that are sufficiently far apart. This term prevented fibers from passing through one another.

Network plasticity was incorporated by having ECM beads within one fiber diameter (200 nm) of each other form new harmonic bonds (Fig. 1c) during cell retraction only. The new harmonic bond had its energy minimum occur when the beads were two fiber radii apart (200 nm). These bonds represented fiber entanglement or crosslinking by permanently changing network connectivity. Non-plastic network simulations omitted bond creation. While the framework allows for a probabilistic bond creation subroutine, bond creation occurred every time two beads contacted one another. A probabilistic bond deletion routine is also possible using this model’s framework but was not explored in this feasibility study.

#### Cell Model

The cell was modeled as a static ellipsoid at the domain center. Each timestep, the cell was defined by 3 semiaxes and their corresponding directions. For initialization, the cell was a 10-µm sphere. ECM beads and segments that overlapped with the initial cell volume were deleted from the system prior to any remodeling and were not replenished as the cell updated its morphology. Throughout the simulation, cell volume was maintained constant.

The model cell interacted with the ECM in four distinct steps: extension, retraction, detachment, and polarization (Fig. 1d). To simulate pseudopod extension, the cell generated forty 2.5-μm spherical tractors 10 μm away from the cell surface, based on experimental pseudopod measurements [27]. Tractor positions were generated in a polarization-dependent manner described in the next section. After generation, the tractors formed harmonic bonds with ECM components within 2.6 μm of the tractor center. The spring constant, K, was the same as above. The rest length, r_0_, was set to 2.6 μm, the sum of the tractor and ECM radii.

During retraction, tractors moved towards the cell surface in fifty 200-nm steps. Retracting tractors also dragged bound ECM towards the cell surface. After each 200-nm tractor displacement step, new crosslinks were allowed to form between contacting ECM beads.

Once tractors reached the cell surface, pseudopod detachment was modeled by deleting tractor-ECM bonds and allowing network relaxation. The resulting network then became the input for the next cycle.

Because of the fixed displacement method of retraction, the model does not have an inherent timescale that it uses to generate traction or relax the surrounding ECM. As such, the 40 tractors generated for each retraction should better be interpreted as the summation of the work the cell inputs into the network given a particular input ECM configuration, rather than independent pseudopod extensions and retractions.

#### Cell sensing of ECM anisotropy and polarization

After the first retraction cycle, the cell updated its morphology by interrogating ECM within 40 μm of its surface. The local ECM structure was represented by the length-weighted fiber orientation tensor, $\boldsymbol{\Omega }$ [27] (Eq. 4),

4 $$\boldsymbol{\Omega }=\frac{1}{\sum_{m}{L}_{m}}\sum_{m}{L}_{m}{{\boldsymbol{n}}}_{{\boldsymbol{m}}}\otimes{{\boldsymbol{n}}}_{{\boldsymbol{m}}}$$

where ${L}_{m}$ and ${{\boldsymbol{n}}}_{{\boldsymbol{m}}}$ are the length and direction vector of the m-th fiber segment in the ellipsoid 40 μm from the cell’s surface.

Cell morphology was informed by matrix anisotropy attenuated or amplified by a power law with a sensitivity parameter, *s*. The normalized cell orientation tensor **Χ** (Eq. 5) is

5 $$\boldsymbol{\rm X}=\frac{{{\boldsymbol{\Omega}}}^{s}}{trace\left({{\boldsymbol{\Omega}}}^{s}\right)}, where {{\boldsymbol{\Omega}}}^{s}\equiv {\boldsymbol{V}}\left[\begin{array}{ccc}{({\Omega}_{1})}^{s}& 0& 0\\ 0& {({\Omega}_{2})}^{s}& 0\\ 0& 0& {({\Omega}_{3})}^{s}\end{array}\right] {{\boldsymbol{V}}}^{{\boldsymbol{T}}}$$

**Fig. 2:**
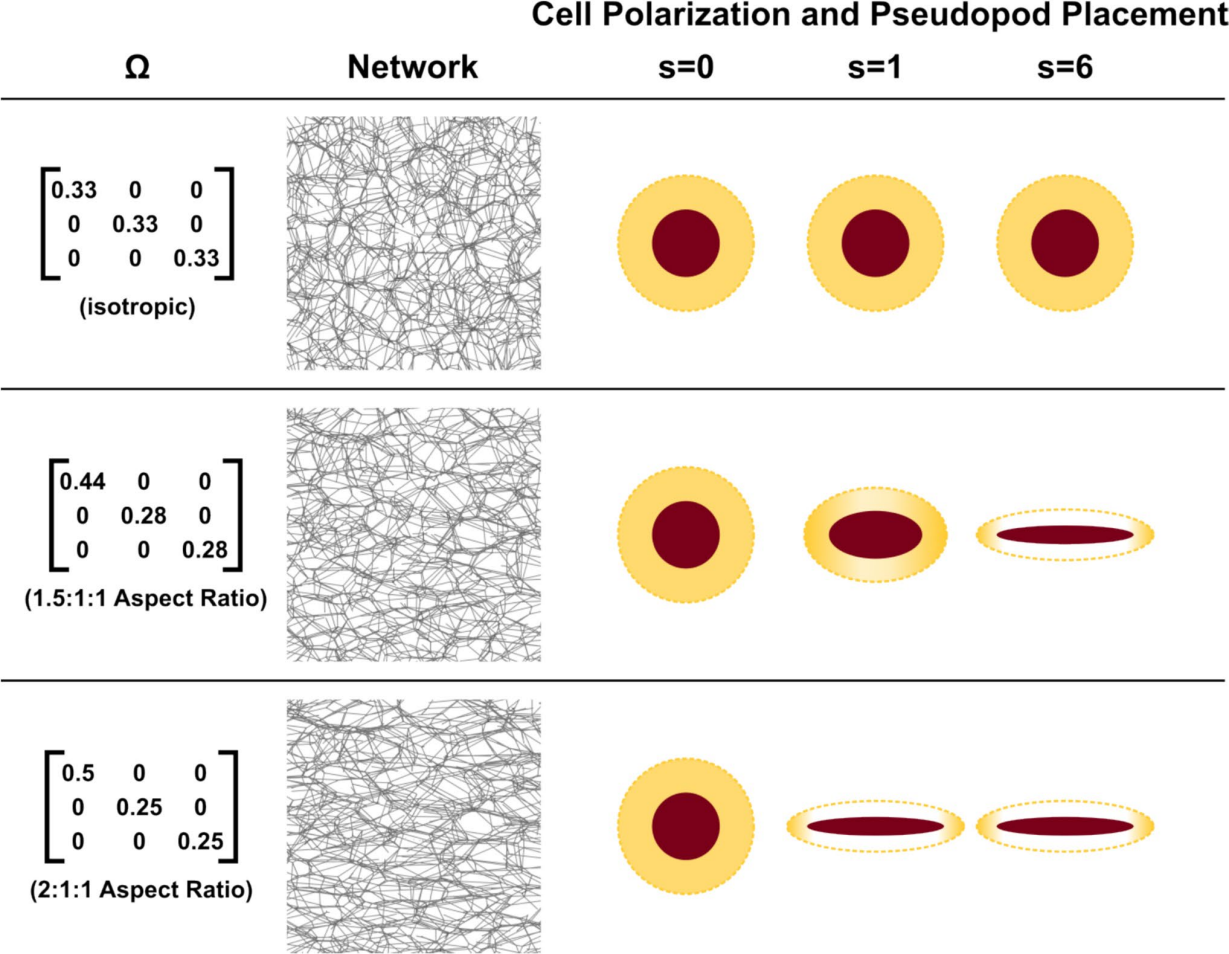
Examples of different network orientation states and resulting cell morphologies.

Where ${\boldsymbol{\Omega}}$ is the fiber orientation tensor for network within 40 μm of the cell surface, **V** is the matrix whose columns are the eigenvectors of ${\boldsymbol{\Omega}}$, and ${({\Omega}_{i})}^{s}$ is the i-th eigenvalue of ${\boldsymbol{\Omega}}$ raised to the *s* power [16]. The sensitivity parameter *s* attenuated or amplified the cell’s response to local anisotropy. Insensitive cells (*s* = 0) maintained spherical morphology regardless of local ECM anisotropy, simulating cells incapable of responding to structural anisotropy and representing a potentially non-physiological lowest limit of ECM-mediated sensing. Low-sensitivity cells (*s* = 1) adopted one-to-one anisotropy with their local environment, and high-sensitivity cells (*s* = 6) polarized strongly even with low amounts of fiber anisotropy (Fig. 2).

The eigenvalues and eigenvectors of **X** determined the relative lengths and directions of the cell ellipsoid’s semiaxes. The cell ellipsoid was scaled to maintain a constant cell volume. A maximum aspect ratio of 5:1:1 was enforced to prevent overly distended cell morphologies.

The cell ellipsoid was then used to generate tractor positions. A second ellipsoid was defined by squaring the lengths of the cell ellipsoid. Forty tractor positions were generated uniformly on this second ellipsoid. Tractors were then projected back to the cell ellipsoid, defining the rest position for each tractor. Initial tractor positions were then defined by displacing the tractors 10 μm from the cell surface along the vector between the cell ellipsoid center and the tractor’s rest position. This schema resulted in spherically uniform tractor placement when the cell ellipsoid was spherical and biased placement in ellipsoidal cells (Fig. 2, gold shaded regions), mimicking directionally mediated pseudopod extension [30].

#### Boundary conditions

The simulation contains 4 types of dynamic beads: ECM beads on the fixed boundary domains, the remaining ECM beads of the network, initial crosslinks between fibers, and the tractors. Beads within 3 μm of the edge of the simulation sphere were fixed and were incapable of forming proximity-based bonds with other fibers. Initial crosslinks in the network participated in harmonic bonds with other ECM beads. These beads were allowed to form new crosslinks with other ECM beads and were able to bind to tractors, but they did not participate in WCA potentials to prevent instabilities during energy minimization. All other ECM beads were subject to harmonic bonds with other ECM beads informed by an adjacency list in the simulation, and proximity-based WCA potentials. These beads could also form harmonic bonds with tractors. Tractors moved via fixed displacement, which was achieved by fixing their position during each minimization step during retraction. As such, while they could participate in harmonic bonding with other ECM types, they remained static during the equilibration process.

The volume of the computational domain was constant. A spherical domain was used to prevent unequal distances between the static cell center and the beads fixed at the simulation volume’s periphery.

#### Mechanical equilibrium

The ECM was assumed to equilibrate faster than the cellular processes of repolarization and tractor extension. Thus, the system was equilibrated after each dynamic process by minimizing the energy of all bead interactions (Eq. 6) described

6 $$\begin{array}{cc}E\left({r}_{1},{r}_{2},\dots ,{r}_{N}\right)=& {\sum}_{i,j}^{N}{E}_{pair}\left({r}_{i},{r}_{j}\right)+{\sum}_{i,j}^{N}{E}_{bond}\left({r}_{i},{r}_{j}\right)\\ & \end{array}$$

where r is the position of any of the N beads in the system, and E represents the pair and bond energies calculated for each pair of beads i and j. Energy was minimized according to a Hessian-free truncated Newton (HFTN) algorithm that iteratively calculated a quadratic approximation of the energy gradient and adjusted bead coordinates until achieving either an energy tolerance, a force tolerance, or calculation maximum detailed in the following section. During the fifty 200-nm tractor displacements, up to 2,000 calculations were allowed. Network equilibration immediately following tractor attachment, retraction, and detachment allowed a maximum of 1,000,000 calculations, achieving a quasi-static equilibrium. This approach was dampened and did not consider the motion of previous steps.

While the HFTN algorithm was used to solve for multiple small displacement steps, other damped dynamics methods could be used to solve the system, such as a quickmin [31] or fire [32] described elsewhere in the molecular dynamics space. However, as the tractor displacements were prescribed and occurred in small steps, a HFTN approach was used to approximate aa quasi-static equilibrium state without defining a timescale for the minimization.

#### Implementation in LAMMPS

Prior to implementation in LAMMPS, the segmented ECM bead positions, bead types, initial uniform tractor positions, and fifty intermediate tractor displacement locations were generated by a custom python script. The LAMMPS implementation made use of 6 different scripts to conduct the simulation from these inputs. Figure S1 provides a graphical representation of this workflow.

In the first input script, ECM beads and their connectivity list were initialized in LAMMPS, forming the network. Non-crosslink ECM beads were relaxed using LAMMPS’ soft pair-wise interaction, preventing beads from overlapping. Existing crosslinks that may have been doubled during the segmentation process were bonded to one another with a short high energy bond, fusing the two beads. The network was minimized using a HFTN minimization with an energy tolerance of 1e−3 or a step limit of 1000.

The updated bead positions were then passed into an initial equilibration script. The soft pair-wise interaction was changed with the previously described WCA pairwise interactions. The network was then minimized using a HFTN minimization with a 1e−10 energy tolerance, 1e−3 force tolerance or a 1,000,000-calculation limit. This input relaxed the network with the inclusion of WCA pairwise interactions and became the input network for the tractors to bind the ECM.

In the third script, the network formed harmonic bonds with tractor beads in the network. All eligible ECM beads, even initial crosslinks, were allowed to form harmonic bonds with overlapping tractors. The updated connectivity list was then recorded for the next input file without minimization.

The contraction input file conducted the displacement-controlled retraction of the tractors towards the cell surface. First, the network was minimized with 1e−10 energy, 1e−3 force, and 1,000,000 calculation tolerances, relaxing the network with the new tractor-ECM bonds. The script then started the displacement-controlled retraction by updating the tractor positions to their intermediate locations and minimizing the network with a 1e−6 energy tolerance, 1e−3 force tolerance, and 2000 calculation limit. After each minimization, bonds between contacting ECM beads were allowed to form. This process repeated 50 times for each intermediate tractor position until the tractors reached the surface of the cell. Finally, the network underwent a longer HFTN minimization with 1e−10 energy, 1e−3 force, and 1,000,000 calculation tolerances, also allowing for new ECM-ECM bonds to form.

The following script simulated cell detachment by deleting tractor bonds from the simulation and minimizing the network with 1e−10 energy, 1e−3 force, and 1,000,000 calculation tolerances. The resulting network was then passed to a python script where the current cell morphology was used to interpret the network 40 μm from the cell surface to calculate the cell’s new morphology, 40 new tractors, and the tractors’ 50 intermediate positions.

The new tractor positions were then input into a final script, which allowed tractors to form new tractor-ECM bonds, but did not undergo minimization. The new connectivity list was then input into the contraction input file until the simulation was completed.

### Simulations

#### Case studies

The effect of plasticity was measured first by simulating cell compaction in isotropic networks with and without ECM-ECM plastic interaction. Next, isotropic networks were generated using Delaunay and Voronoi tessellations, remodeled by insensitive, low-sensitivity, and high-sensitivity cells, and then analyzed for markers of remodeling. Patterns of heterogeneous remodeling were further analyzed in plastic Voronoi networks remodeled by cells of varying sensitivity. Finally, the interaction between pre-existing network alignment and sensitivity was examined by simulating cells of different sensitivity remodeling Voronoi networks with increasing initial alignment. All simulations were repeated 10 times for each combination of cell sensitivity and pre-existing alignment.

#### Analysis of results

The distributions of initial fiber length were recorded for all generated isotropic Voronoi, aligned Voronoi, and isotropic Delaunay networks. The distribution in node connectivity was also measured for Delaunay networks. Voronoi network connectivity was not recorded as each node always has a connectivity of 4.

During analysis, the ECM was sectioned in two different ways: shells and conical ellipsoidal sectors. For shells, the ECM was sectioned into 5 μm-thick ellipsoidal shells a distance away from the cell surface. This analysis provided aggregate data as a function of distance from the cell surface (ρ). Conical ellipsoidal sectors were constructed by dividing shells based on θ, the network’s elevation angle with respect to the cell’s principal axis, in increments of π/32.

ECM remodeling was quantified for both segmentation methods. ECM densification after cell detachment was measured by calculating fiber volume fraction (Eq. 1). ECM radial reorientation after detachment was measured by calculating the orientation tensor (Eq. 4), but using spherical coordinates to measure the relative proportion of fibers oriented in the radial, polar, and azimuthal directions. Bond strain was measured by averaging individual ECM bond strains at peak retraction and after detachment. The angular heterogeneity of each metric was calculated by fitting the directional data to a von Mises distribution, providing μ (the central angle) and κ (the concentration parameter), for the distribution.

Fiber-level data was also visualized by measuring the strain within fiber segments during cells’ 1^st^, 10^th^, and 40^th^ retraction. Histograms of the fiber strains were generated and the mean fiber strain was reported. A visualization of fiber strains was also generated by plotting high strain segments during the 10^th^ and 40^th^ retractions. Cell interaction with the ECM was quantified by measuring the number of tractors that formed bonds with the ECM, the amount of ECM beads that each tractor bound, and the count of ECM beads in the tractor placement zone.

Cell polarization over the 40 retractions was quantified as the ratio between the cell’s largest and smallest semiaxes. Polarization direction was given by the eigenvector associated with the largest semiaxis.

Statistical analysis was conducted using one- and two-way ANOVA. Post-hoc multiple comparisons were analyzed using Tukey’s HSD test. Significance was determined at *p*-values < 0.05.

### Cell culture and imaging

Normal Human Dermal Fibroblasts (nHDFs) purchased from Lonza (Basel, Swizterland, Catalog no.: CC-2511) were stained with CellTracker Orange (Thermo Fisher, Waltham, MA) following manufacturer specifications to stain the cell cytoplasm. Following 24-hour incubation on TC plastic, cells were then seeded in 1 mg/mL rat-tail collagen gels at 37C following the same procedure as previously published work [11]. After 30 minutes, culture media containing DMEM, 10% FBS (v/v) and 1% Penn-Strep (v/v) was added to the culture wells. Cells were then incubated at 37 C and 5% CO_2_ for 18 hours. After, cells were fixed with 4% (v/v) paraformaldehyde solution in DPBS prior to imaging.

Fixed cells were imaged using a Zeiss LSM 510 microscope using a 40 × , 1.2 NA water immersion objective. Cells were imaged in z-stacks using a bottom-up approach. Fluorescence microscopy was used to image the CellTracker dye and confocal reflectance was used to image the collagen network surrounding the cell. Images were then filtered with a median filter before being maximum projected in the z-axis.

## Results

The following results illustrate the platform’s ability to effect different patterns of remodeling, network heterogeneity, and cell response in networks across a limited parameter set including network plasticity, initial network connectivity, cell sensitivity to the ECM, and pre-existing alignment in the ECM.

### Effect of Plasticity and Topology

**Fig. 3:**
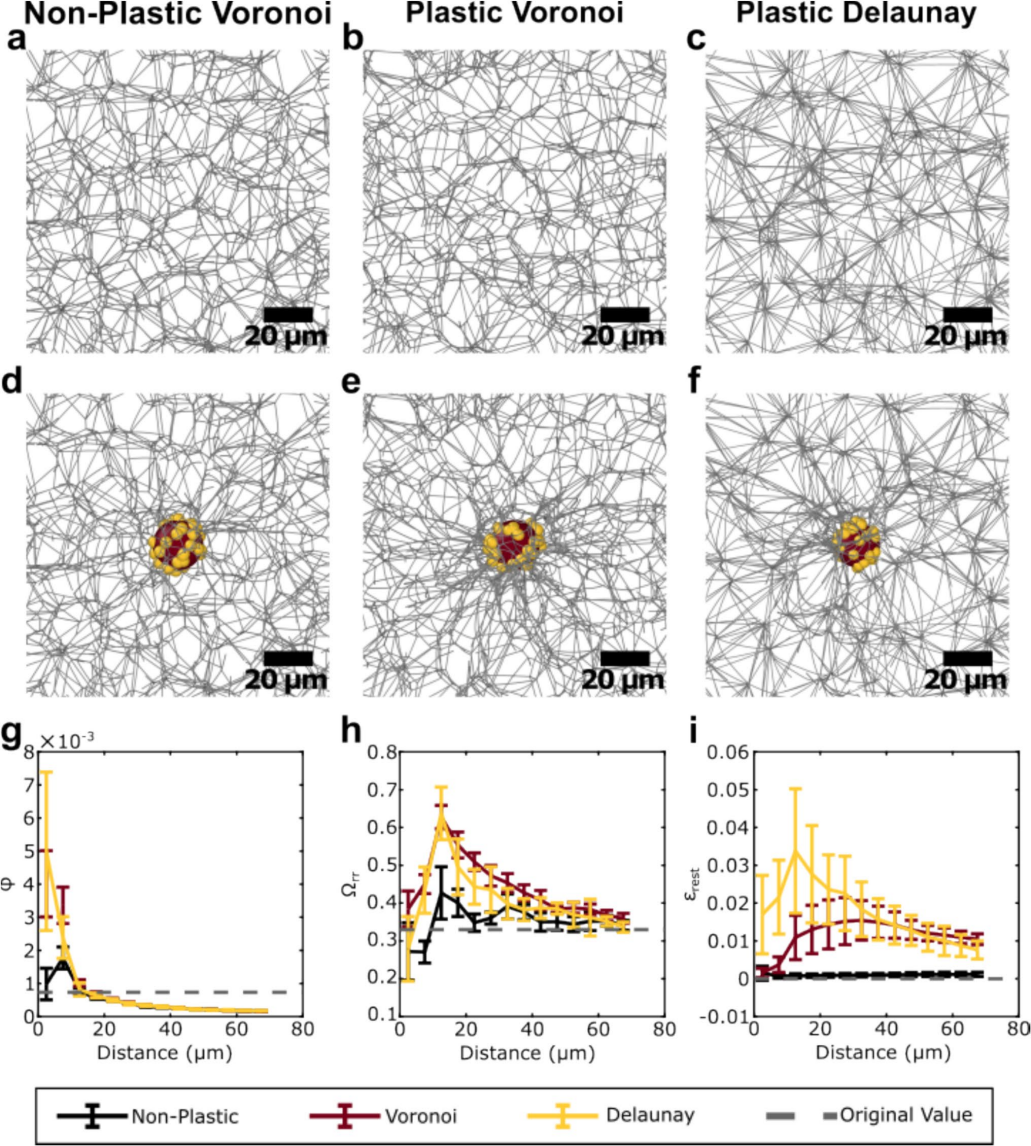
**a** Initial and (d) final ECM configuration of non-plastic network after 30 retractions. **b**, **e** Same for plastic Voronoi network. **c**, **f** Same for plastic Delaunay network. Cell centers are shown in maroon and tractors in gold. **g** Fiber density increases near the cell, especially in plastic simulations. Cell centers are shown in maroon and tractors in gold. **h** Results for radial fiber reorientation. **i** Results for bond stretch. Error bars show standard deviation. Distances on the x-axis denote distance from the cell surface**.** n = 10 for each case.

To measure the effect of plasticity and network topology on ECM remodeling, non-plastic Voronoi, plastic Voronoi, and plastic Delaunay networks were simulated with insensitive cells (Fig. 3a-c). Isotropic Voronoi networks had a median fiber length of 5.92 μm and a mean length of 15.86 (Fig. S2a). All initial crosslinks had a connectivity of 4. The isotropic Delaunay networks had a median fiber length of 27.52 μm and a mean length of 36.68 μm (Fig. S2b). Crosslinks in the Delaunay networks had a median connectivity of 15 and a mean connectivity of 15.55 (Fig. S2c). These results indicated that while the different networks had similar fiber volume fraction, Voronoi networks were typified by both shorter fibers and a looser connectivity than their Delaunay counterparts.

After 30 retractions (Fig. 3d-f), all three network types exhibited elevated fiber density (Fig. 3g), with a smaller effect in the non-plastic network. The difference between the non-plastic and plastic Voronoi networks was significant within 5 μm of the cell. Compared to the plastic Delaunay network, the plastic Voronoi network had significantly higher fiber density between 5 and 30 μm. Radial fiber reorientation was observed in all networks (Fig. 3h), but the effect was smallest in non-plastic networks. Plastic Voronoi networks were significantly more radially oriented than non-plastic Voronoi networks up to 60 μm away. Plastic Voronoi networks also had significantly greater radial orientation than plastic Delaunay networks between 20 and 45 μm. Bond strain (Fig. 3i) after detachment was nearly 0 for the non-plastic networks but was elevated in both plastic networks. Compared to the non-plastic network, the plastic Voronoi network had significantly higher resting bond strain at distances greater than 15 μm. Between the plastic networks, the plastic Delaunay network had significantly higher bond strains up to 30 μm away and significantly lower strains at distances greater than 65 μm away. The differences observed between the Voronoi and Delaunay networks are likely driven by the latter network’s greater initial connectivity (nodal degree = 4 vs 15.5). The greater connectivity results in bond creation between already highly connected segments, conferring additional “tightness” to the system, particularly near the cell surface.

These results were consistent with previous findings on network connectivity [20, 33]. Because Voronoi networks better match the loose connectivity of collagen networks, plastic Voronoi networks were used in all subsequent simulations.

### Model validation: ECM remodeling

**Fig. 4:**
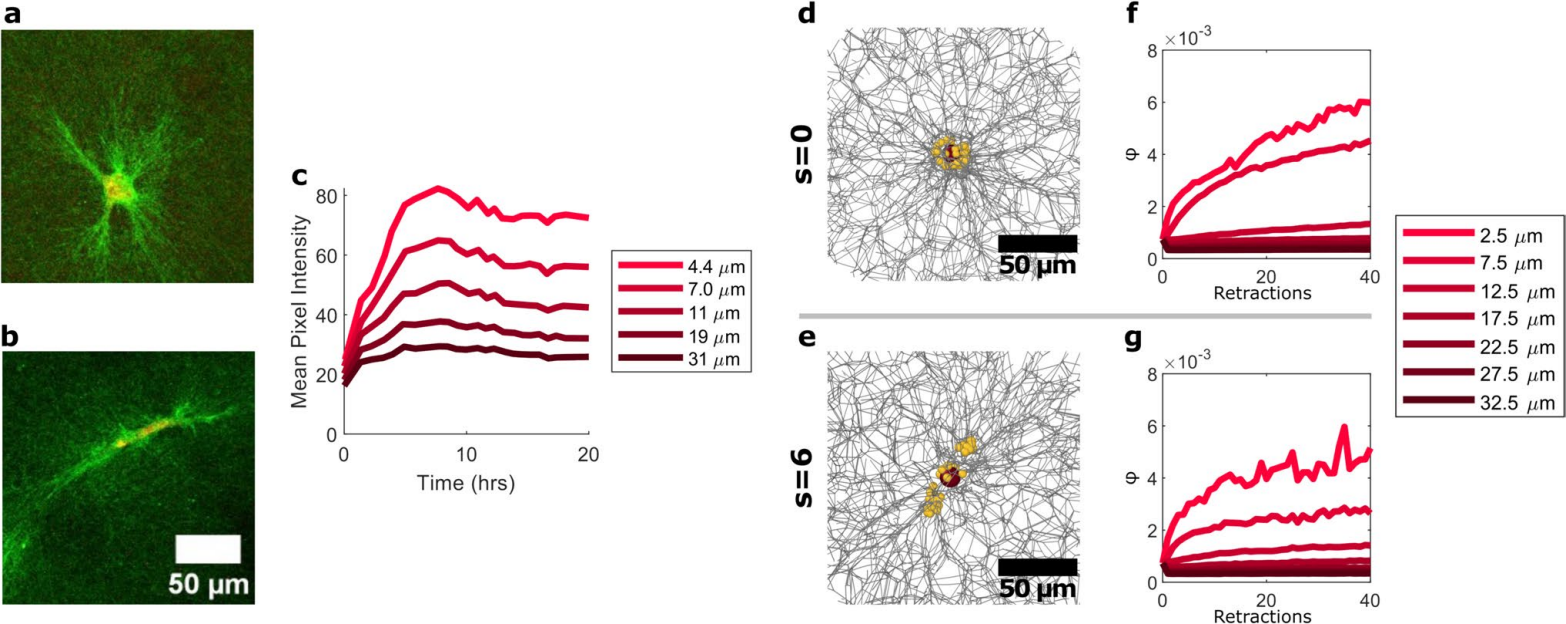
**a**, **b** nHDFs remodeled initially isotropic collagen gels over 18 hours. Experiments were performed by AWYL during a visit to the Genin lab at Washington University, following the methods of [11]. **c** Remodeled networks exhibited plateauing ECM densification vs time which also decreased with distance from the cell (data from [11]). **d**, **e** Simulated networks exhibit radial or linear fiber realignment depending on sensitivity. Cell centers are shown in maroon and tractors in gold. **f**, **g** Mean network densification was highest near the cell for both insensitive and sensitive cells; n = 10 for each case.

Simulations of cells compacting isotropic gels were performed for comparison with available experimental data [11]. Previous experiments in collagen gels demonstrated that cells generate heterogeneous microenvironments, both in terms of fiber alignment (Fig. 4a,b) and density (Fig. 4c). Regions closest to the cell densify considerably more than farther regions, and that densification plateaued with time.

*In silico*, cells of different sensitivity were simulated remodeling initially isotropic fiber networks. After 40 retractions (Fig. 4d,e), networks demonstrated visible radial and linear realignment, with lower sensitivity cells exhibiting more radial alignment and higher sensitivity cells exhibiting more linear alignment. Fiber density in the ellipsoidal shells near the cell initially increased quickly, but with decreasing densification rate (Fig. 4f,g), consistent with experiments. The densification in each shell decreased with distance from the cell. These trends were observed across all sensitivities (*s* = 0, 1, 2, 4, 6).

### Spatial variation in network remodeling

#### Network densification

**Fig. 5:**
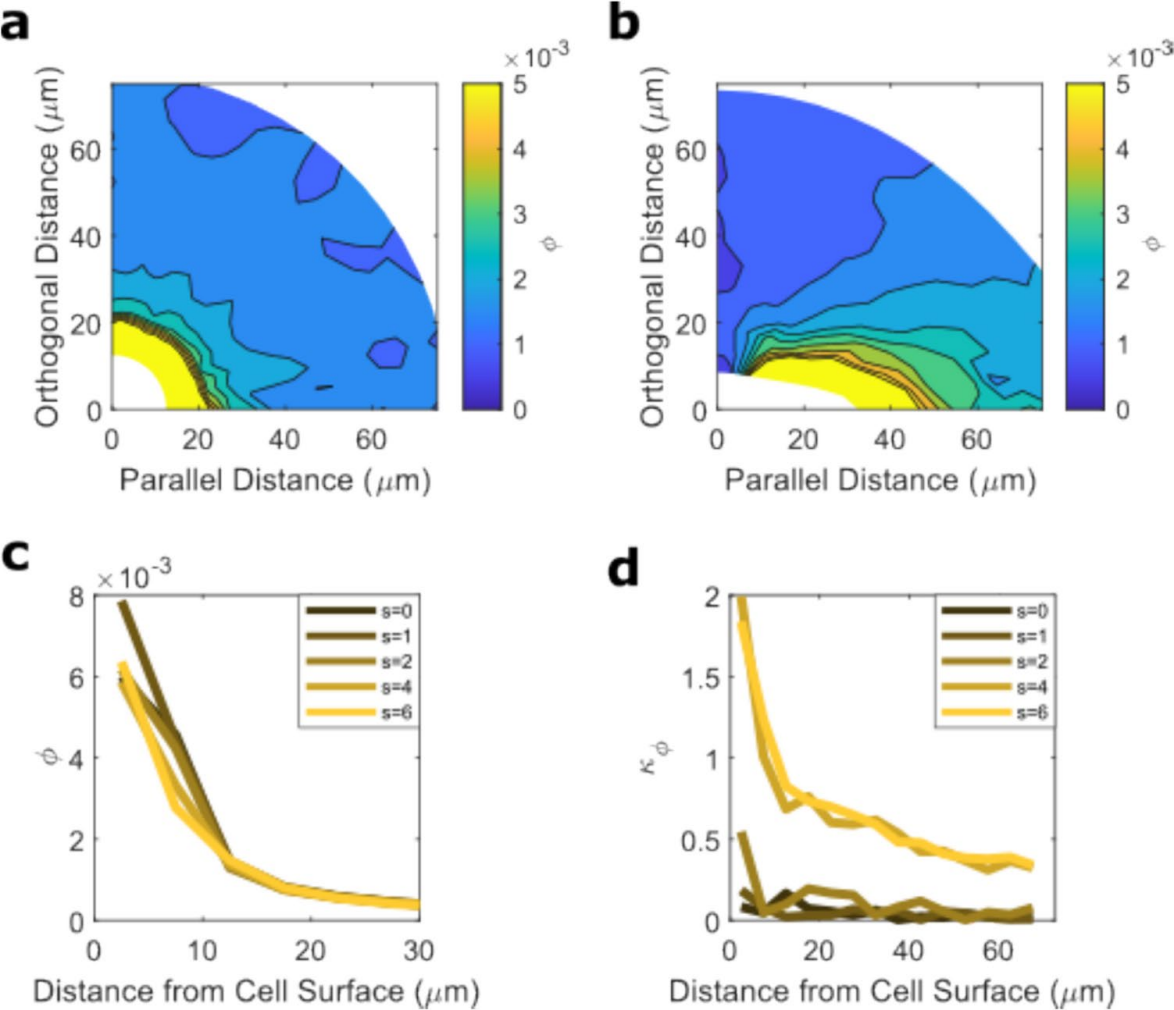
**a** Distance and angular dependence of the mean fiber volume fraction at *s* = 0. **b** Same for *s* = 6. **c** Mean shell fiber volume fraction vs distance from the cell for different sensitivities. **d** κ parameter vs distance from the cell for different sensitivities. Network density heterogeneity increased with both sensitivity and proximity to the cell. n = 10 for all cases.

Contour plots of the network density at *s* = 0 and 6 demonstrate that network densification depends on both distance and angle from the cell’s primary axis (Fig. 5a,b). Across all sensitivities, final network density was higher in ellipsoidal sectors near the cell. Higher sensitivity cells also exhibited angular heterogeneity in network densification, with higher densification along the cell’s primary axis. Though the pattern of densification across sectors was affected by cell sensitivity, aggregate ECM densification measured in the full shell was not significantly affected (Fig. 5c). Across all sensitivities, the von Mises concentration parameter κ was highest in shells closer to the cell surface, indicating greater volume fraction heterogeneity. Further, κ increased with sensitivity, indicating that increased cell sensitivity resulted in more heterogeneous network remodeling.

#### Network radial reorientation

**Fig. 6:**
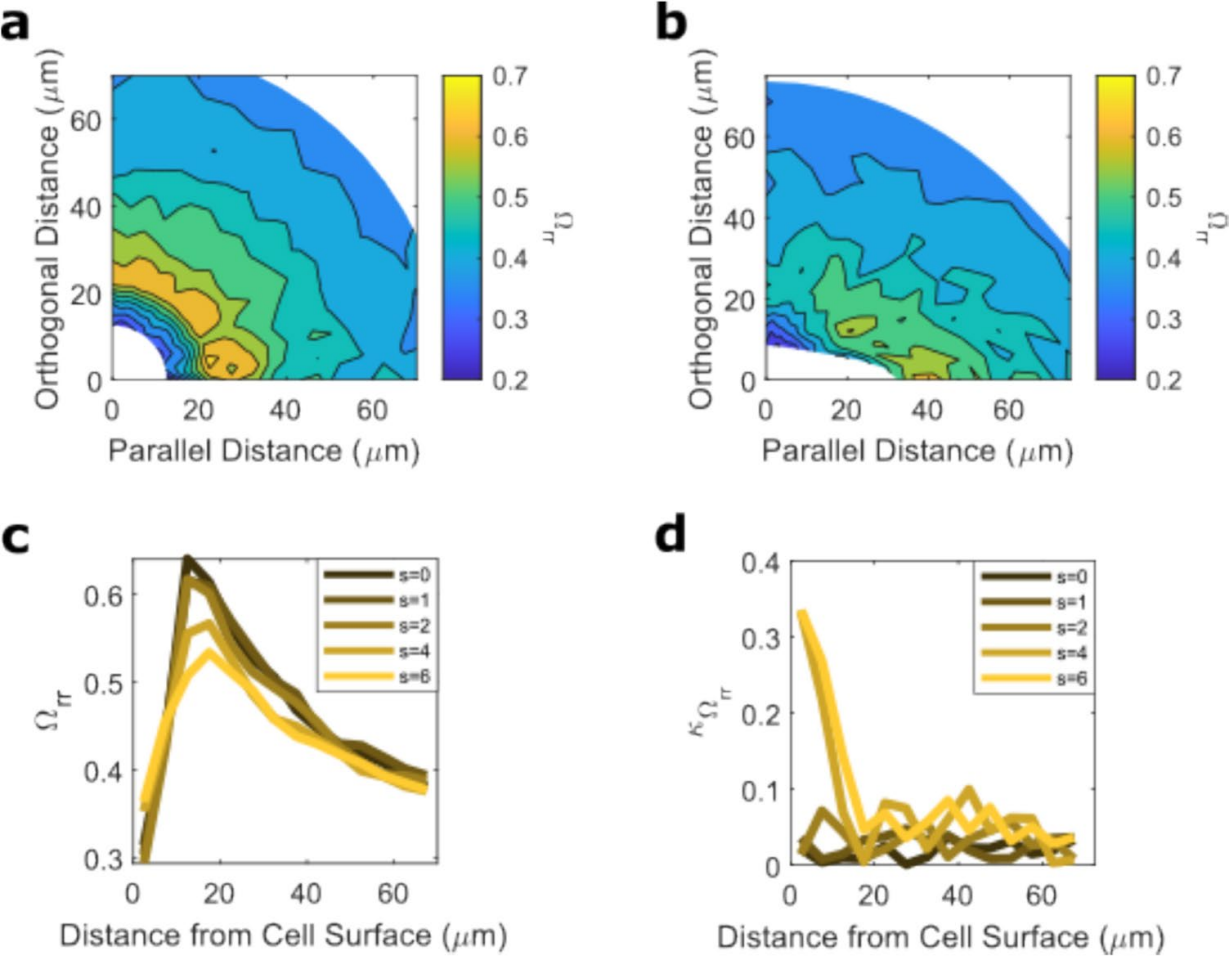
**a** Distance and angular dependence of the mean radial component of the fiber orientation tensor for *s* = 0. **b** Same for *s* = 6. **c** Radial alignment decreases with greater sensitivity. **d** Concentration parameter (i.e. alignment heterogeneity) increased in sensitive cells, but only near to the cell surface. n = 10 for all cases.

Network reorientation was analyzed for radial and angular heterogeneity. The radial component of the spherical orientation tensor for insensitive (*s* = 0) and highly sensitive (*s* = 6) cells exhibited heterogeneous patterns (Fig. 6a,b). Unlike the fiber volume fraction, the proportion of fibers aligned radially reached its maximal value 10 to 20 μm from the cell (Fig. 6c), just outside cell tractor range. The increase in radial alignment of the entire shell between 10 and 20 μm was significant compared to regions nearer to and farther from the cell (Fig. 6c). High-sensitivity cells (*s* = 4, 6) had significantly lower radial orientation than both low-sensitivity (*s* = 1) and insensitive cells (*s* = 0). When the fiber orientation distributions at each distance were fit to a von Mises distribution, the concentration parameter κ varied with distance from the cell in highly sensitive cells (Fig. 6d). While the value of κ was higher close to the cell in high sensitivity cells, the lower κ values indicated that the alignment heterogeneity was not as pronounced as fiber density heterogeneity.

#### Network bond strain

**Fig. 7:**
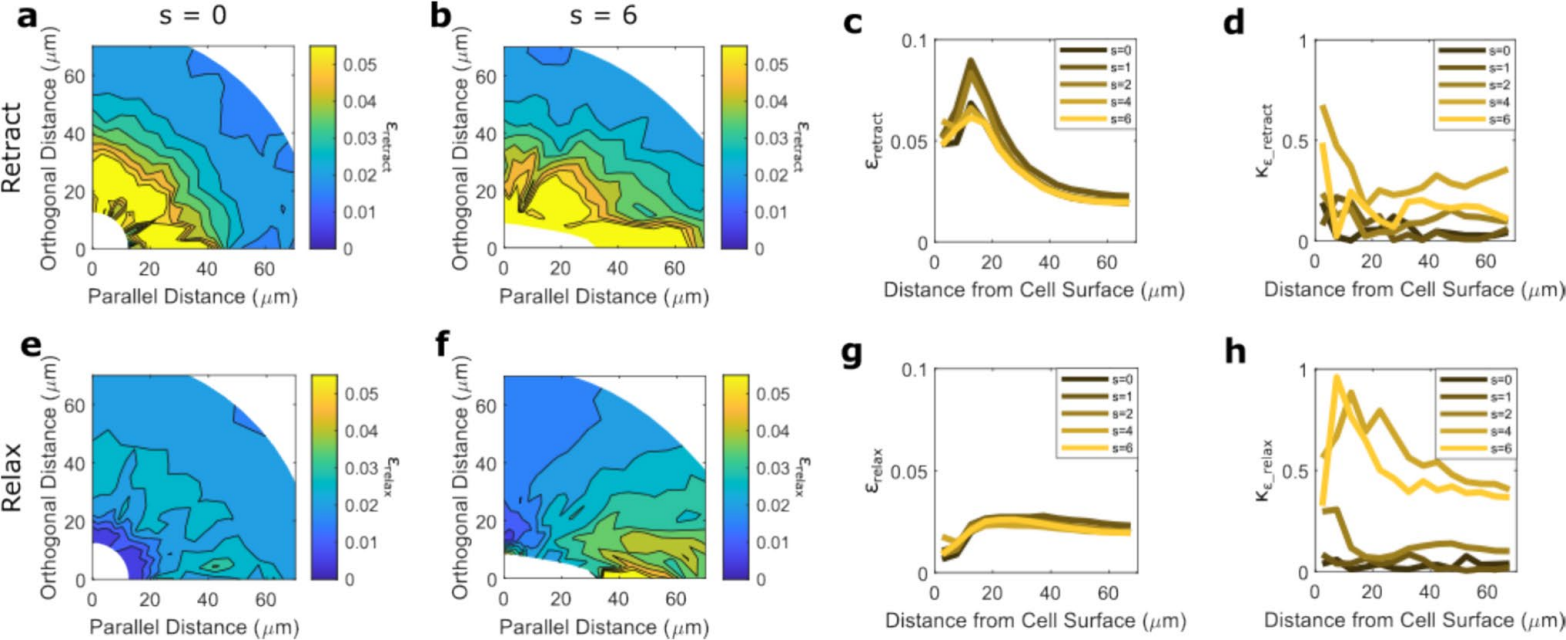
**a** Distance and angular dependence of mean fiber strain during retraction at *s* = 0. **b** Same for *s* = 6. **c** Mean fiber strain during retraction. **d** von Mises κ parameter of mean fiber strain during retraction vs. distance from the cell. **e** Distance and angular dependence of mean relaxed fiber strain for *s* = 0. **f** Same for *s* = 6. **g** Mean relaxed fiber strain vs. distance from the cell. **h** von Mises κ parameter of the fiber strain during retraction at different distances. n = 10 for all cases.

Similar analysis was conducted on fiber strain at peak retraction and release. During peak retraction, fiber strain was highest near the cell surface across all sensitivities (Fig. 7a,b) and exhibited angular heterogeneity in sensitive cells. Average strain increased between the cell surface and 12.5 μm from the cell and then decreased at farther distances across all sensitivities (Fig. 7c). The maximal strains at 12.5 μm were significantly higher than all other positions. Two-way ANOVA determined that both distance from the cell and sensitivity significantly impacted average strain with insignificant interaction. High-sensitivity cells (*s* = 6) generated significantly lower mean bond strains than low-sensitivity cells (*s* = 1 or 2) but were not significantly different from insensitive cells. The von Mises κ parameter from the fitted directional data was elevated with higher cell sensitivity but did not necessarily decrease with distance from the cell surface (Fig. 7d).

The distribution of fiber bond strains after network relaxation exhibited radial dependence across all sensitivities and angular dependence in high-sensitivity cells, with higher strains occurring parallel to cell polarization (Fig. 7e,f). Fiber strains within 7.5 μm of the cell were significantly lower than those farther away (Fig. 7g). Across all distances, there were no significant differences in average strain between different cell sensitivities. Fitted von Mises κ parameters (Fig. 7h) were elevated with increased sensitivity and mostly decreased with distance from the cell. Thus, bond strain heterogeneity increased with both cell sensitivity and proximity to the cell surface, and it was elevated over a larger spatial range in sensitive cells.

#### Fiber strain heterogeneity in isotropic and aligned fiber networks

Over the span of 40 retractions, the amount of force carried in each fiber segment was recorded in low- and high- sensitivity cells in isotropic and aligned (Ω_xx_ = 0.5) networks. Distributions of the initial fiber lengths in isotropic and aligned networks (Fig. S2a) demonstrated that isotropic and aligned networks had median fiber lengths 5.95 and 6.22 μm, respectively. They also had similar mean fiber lengths, 15.86 and 16.27 μm for the isotropic and aligned networks, respectively.

**Fig. 8:**
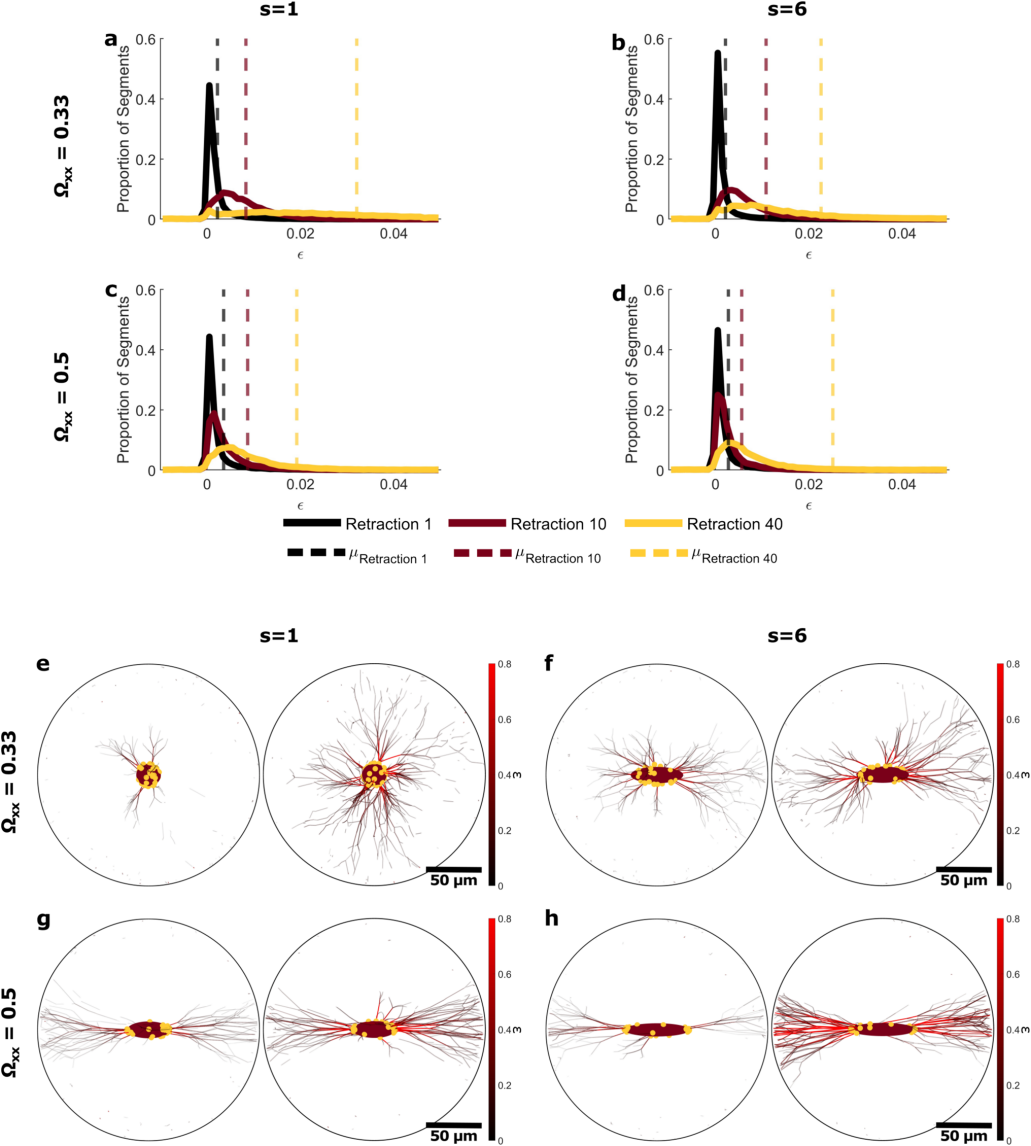
Histograms of segment strains during the 1st, 10th and 40th retraction for representative cases of low sensitivity (**a**) and high sensitivity (**b**), cells in isotropic networks and low sensitivity (**c**), and high sensitivity (**d**) cells in aligned networks showed an increase in fiber strain across all networks. Mean fiber strain at each time point is denoted by a vertical dashed line. The distributions of high-tension fibers in each representative case (**e**-**g**) during the 10th (left) and 40th retraction (right) show a dependence on both cell sensitivity and initial fiber alignment. Networks are rotated with respect to the cell’s principal axes, with the first principal axes being plotted in the x axis. The color and transparency of fiber segments were scaled by their strain. Cell morphology is denoted in maroon and tractors in gold.

Histograms of the forces in each segment during the 1st, 10th, and 40th retraction were visualized for representative cases (Fig. 8a-d). Across all cases, the average fiber strain increased with subsequent retractions. However, the data do not come from a normal distribution, instead exhibiting a long tail. In each sample, the data skews right, indicating that a small number of fiber segments are much higher than the rest of the segments in the distribution.

To better visualize the forces in these fibers, segments were plotted in their retracted state at the 10th and 40th retractions for each representative case (Fig. 8e-h). Generally, the segments that were radially oriented and closer to the cell surface had the greatest tension, with strains much higher than the mean strain in the network. In later retractions, more of the network experienced an elevated tension. The sensitivity of the cell and the pre-existing alignment of the network influenced the spatial pattern of the strain through the network.

In initially isotropic networks, a greater proportion of the network experienced tension during both the 10th and 40th retraction when compared to their aligned counterparts. Lower sensitivity cells generated increased strain in a more homogenous pattern, while their higher sensitivity counterparts primarily generated strains in the direction of their principal axis. In aligned networks, forces were primarily transmitted along fibers aligned in the x-direction. While both high and low sensitivity cells propagated strains in the orthogonal directions at later timepoints, the strains remained more concentrated in the x-direction (Table 1).

**Table 1:** Simulation parameters

Symbol	Value	Description	References
r_col_	100 nm	Radius of collagen fiber	[28]
r_0, fiber_	400 nm	Rest length of collagen harmonic bonds	
K	5000 kcal/nm^2^	Spring constant of fiber-fiber and fiber-tractor harmonic springs	
ε	1	Weeks-Chandler-Anderson scaling coefficient corresponding with well-depth of a Lennard Jones potential energy	
σ	0.0891 nm	The length scale term of the Lennard-Jones energy potential used in the Weeks-Chandler-Anderson potential.	
r_cf_	200 nm	The cut-off length of the Weeks-Chandler-Anderson Potential	[28]
r_tractor_	2.5 μm	Radius of a tractor corresponding to a pseudopod width	[27]

The discrete nature of the tractors and fiber network also caused tractors directly engage with a small proportion of ECM beads in the tractor placement zone. Tractor-ECM interactions were measured by counting the tractors that formed bonds with the ECM, the unique ECM beads that bound to tractors, and the total number of ECM beads in the tractor placement zone. From there, the proportion of the beads in the tractor placement zone could be determined (Table 2).

**Table 2:** Mean measurements of tractor-ECM interactions. Data is grouped by the cell sensitivity, initial ECM alignment, and retraction number. Reported data includes the mean number of tractors bound to the ECM, the mean number of bonds each tractor formed, the mean number of unique ECM beads bound to tractors, and the average number of ECM beads in the tractor placement zone. Standards of deviation are presented for each value in parentheses. n = 10 for each measurement.

Sens	Ω_xx_	Retraction	Mean bound tractors	Mean bonds per tractor	Mean bound ECM beads	Mean ECM beads in placement zone	Mean proportion of bound ECM
1	**.33**	**1**	15.44 (5.66)	7.13 (6.78)	87.89 (56.12)	2223.44 (366.70)	0.0400 (0.0245)
		**10**	15.67 (3.57)	8.08 (8.71)	105.00 (58.74)	4677.67 (349.22)	0.0225 (0.0130)
		**40**	24.56 (7.42)	22.11 (29.98)	435.33 (323.51)	7637.11 (1936.42)	0.0532 (0.0346)
	**.5**	**1**	15.80 (3.46)	9.94 (10.59)	130.50 (62.01)	2338.70 (317.64)	0.0557 (0.0228)
		**10**	28.30 (4.40)	19.80 (21.02)	318.70 (129.41)	4192.30 (732.60)	0.0753 (0.0227)
		**40**	33.20 (3.61)	57.91 (105.54)	998.20 (580.31)	8002.70 (1657.19)	0.1206 (0.0449)
6	**.33**	**1**	16.60 (3.92)	8.25 (9.30)	113.30 (80.42)	2277.80 (361.66)	0.0489 (0.0308)
		**10**	28.90 (4.28)	24.73 (24.78)	370.20 (142.47)	4728.10 (810.74)	0.0776 (0.0252)
		**40**	32.50 (3.63)	36.08 (40.39)	628.00 (216.65)	7289.90 (1425.82)	0.0857 (0.0250)
	**.5**	**1**	23.25 (8.46)	16.27 (12.38)	177.63 (82.47)	2435.88 (318.92)	0.0594 (0.0306)
		**10**	31.00 (5.71)	40.57 (45.57)	395.75 (150.64)	3938.75 (528.07)	0.1013 (0.0363)
		**40**	36.38 (3.16)	79.61 (116.92)	1082.75 (425.25)	7386.75 (1259.71)	0.1440 (0.0481)

Broadly, the number of tractor-ECM interactions increased with the number of retractions, as measured by both the number of tractors forming bonds with the ECM and the number of unique ECM beads binding to tractors by the end of each retraction. Further the number of bonds each tractor formed with the ECM increased with retraction number. Finally, the proportion of the network that was directly bound to tractors at the end of each retraction increased with the number of retractions. Together, these results indicate that as the network densified around the cell, the number of tractor-ECM interactions and the proportion of the network that bound to the tractors increased with subsequent retractions.

### Spontaneous polarization

#### Cell polarization versus time

**Fig. 9:**
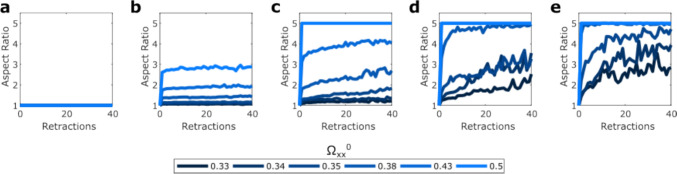
Mean cell aspect ratio (n = 10) vs retractions, **a** *s* = 0, **b** *s* = 1, **c** *s* = 2, **d** *s* = 4, **e** *s* = 6. Line color corresponds to initial network x-alignment as measured by the xx-component of the fiber orientation tensor, Ω_xx_. Dark lines correspond to unaligned networks, and light blue lines correspond to strongly aligned networks.

Over 40 retractions in the simulation, the ratio between the cell’s longest and shortest axes was computed. As expected, insensitive cells (Fig. 9a) maintained a spherical morphology throughout the simulation. For sensitive cells in isotropic networks (Fig. 9b-e, dark blue), cell aspect ratio increased over time. Cells with higher sensitivity exhibited both increased polarization rate and higher final polarization. Although networks had no strong initial alignment, multiple retraction cycles increased local fiber alignment, increasing cell polarization. When initial pre-alignment was introduced (Fig. 9b-e, light blue), polarization was amplified across all sensitivities.

#### Final polarization magnitude and direction

**Fig. 10:**
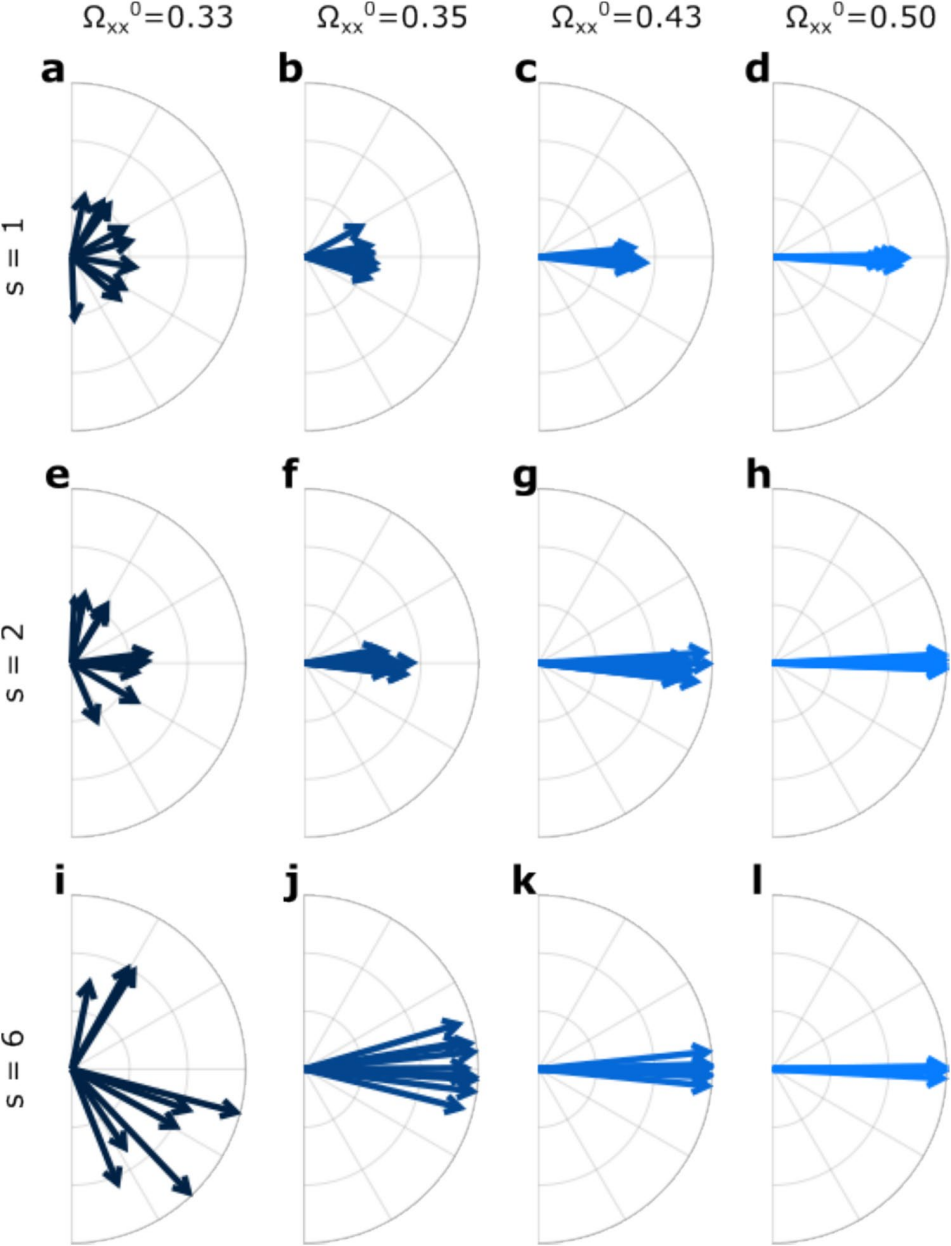
The direction of each cell’s major axis in the x-y plane plotted vs the cell’s longest axis after 40 retractions (n = 10). Each arrow represents a different cell among 10 different runs. Sensitivities: (a-d) *s* = 1, (e-h), *s* = 2, (i-l) *s* = 6. Initial ratio of fiber alignment in x, y, z coordinate directions: (a,e,i) 1:1:1. (b,f,j) 1.08:1:1, (c,g,k) 1.5:1:1, (d,h,l) 2:1:1. Dispersity decreased with both sensitivity and initial alignment.

The lengths of individual cells’ major semiaxes were plotted against their orientation in the x-y plane. In unaligned gels, cells across all sensitivities s > 0 increased in semimajor axis length (Fig. 10, first column), but the polarization direction was random. Thus, sensitivity increased the magnitude of cell polarization in initially unaligned gels, but it did not predispose cells to orient in a particular direction. When pre-existing alignment was introduced into the networks, cells of non-zero sensitivity aligned in the x-direction. Cell sensitivity increased both the degree of polarization and directional preference.

### Effect of pre-alignment

**Fig. 11:**
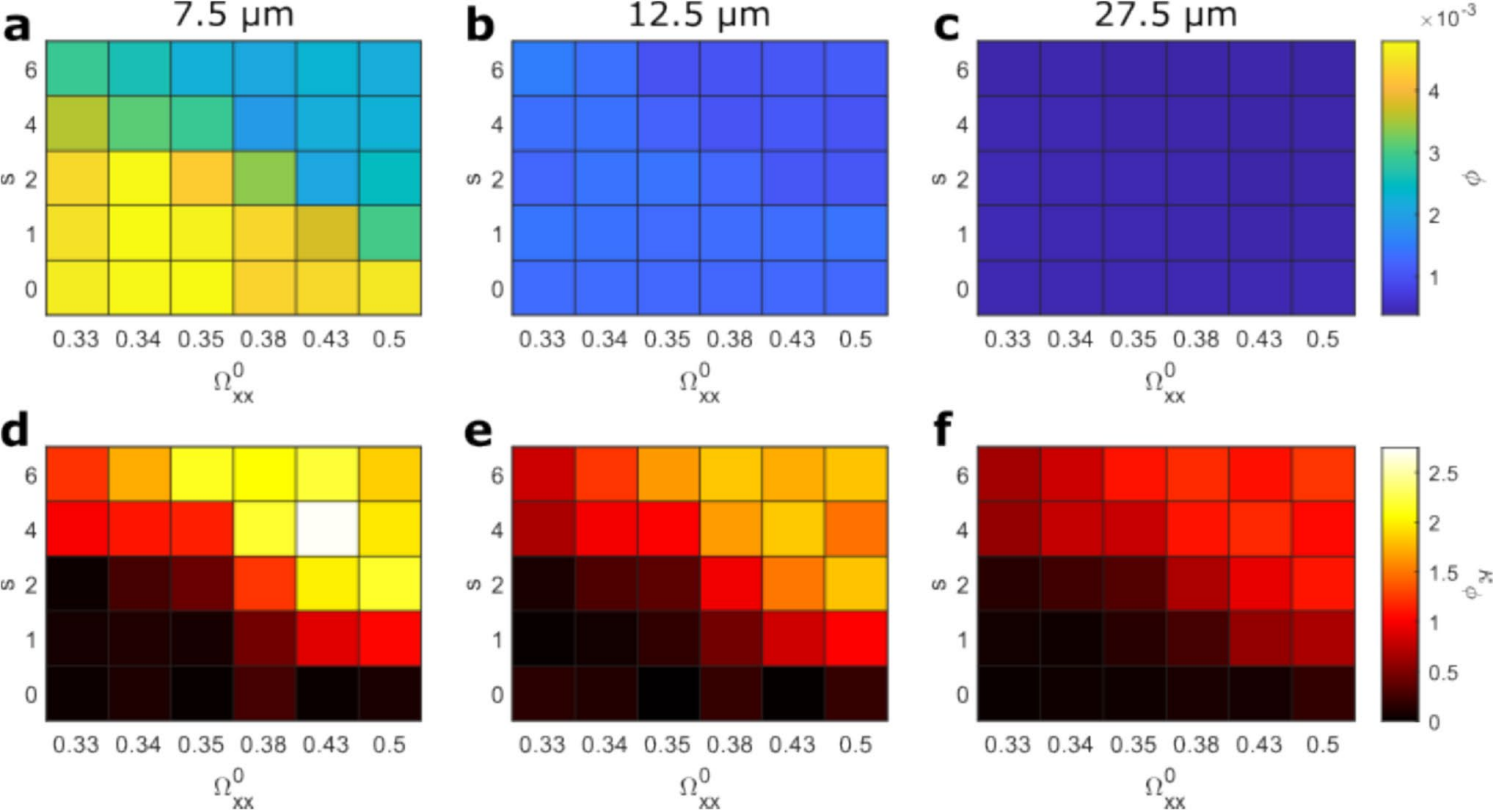
**a**–**c** Average fiber volume fractions (n = 10) after 40 retractions plotted against the initial network x-alignment and cell sensitivity. **a** 7.5 μm, **b** 12.5, and **c** 27.5 μm from the cell surface. **d**–**f** Fitted von Mises κ parameters for fiber density plotted against the initial network x-alignment and cell sensitivity (**d**) 7.5 μm, **e** 12.5, and **f** 27.5 μm from the cell.

Aggregate ECM densification was quantified at different distances for different combinations of cell sensitivity and network pre-alignment. Near the cell (Fig. 11a), densification decreased with increased pre-alignment and cell sensitivity. Intermediate distances (Fig. 11b) exhibited a similar densification pattern with reduced magnitude, but farther distances (Fig. 11c) did not.

Directional data at each distance were fitted with von Mises distributions. Near the cell (Fig. 11d), the κ parameter increased with both sensitivity and network pre-alignment. Farther from the cell (Fig. 11e,f), the same correlation emerged but with lower magnitude. Thus the heterogeneity signal remains Ω- and s-dependent at further distances while the aggregate effects decay.

## Discussion

By incorporating elements of cell-ECM sensing and ECM plasticity, the model presented herein recapitulates certain experimentally-observed trends in cell-mediated ECM remodeling. The model exhibits a distance-dependent densification pattern that decreases in rate with subsequent retraction cycles, qualitatively matching published experimental results [11]. The cell model also captures long-observed trends of cell polarization behavior: persistent polarization [34] and strong polarization responses in anisotropic 3D ECMs [4, 30]. Qualitative observations of remodeled ECM indicate that fibers near the cell reorient either radially about the cell center or linearly with respect to the cell’s polarization direction [4, 35]. The model reproduces this trend, with less sensitive cells exhibiting greater radial fiber reorientation and more sensitive cells exhibiting greater linear fiber reorientation. These combined trends of increasing density and linear alignment are often associated with network stiffening or mechanical anisotropy [36]. Thus, the ability to examine both trends separately may provide further insight into microstructural mechanical anisotropy. Further, the model enables investigation of the effect of existing ECM alignment on remodeling and ECM strain. Previous simulations in discrete networks have demonstrated that a small proportion of fibers are loaded during cell retraction [24, 25] and that these fibers can transmit forces across long distances [25]. The model recapitulates this trend and demonstrates that directional strain fields arise in both initially isotropic ECM networks remodeled by high-sensitivity cells or less sensitive cells in aligned networks. Experimentalists have developed numerous ways of creating aligned fiber networks for applications from simulating the tumor microenvironment [37, 38] to developing regenerative medicine platforms [39]. Such advances make it essential that models incorporate a cell-ECM sensing mechanism to investigate how remodeling patterns change with either pre-existing or evolving anisotropy.

The current modeling approach includes simplifications that may warrant future investigation. The boundary conditions of the model may make the results sensitive to edge effects. In experiments, collagen gels are observed to undergo large amounts of compaction [40]. Currently, the model maintains a constant volume with fixed boundaries, and as a result it may not be suitable for simulating long-term remodeling behavior or highly cellularized tissues. This simplification in the boundary conditions may also prevent good quantitative matches for both the ECM network densification and realignment, as fibers nearer to the boundaries are unable to displace towards the cell or propagate stresses beyond the simulation volume. Additionally, previous simulations in discrete fiber networks have demonstrated that a small proportion of fibers transmit significantly higher forces across long distances [24, 25]. Especially in fibrous environments, non-affine deformations are often necessary for recapitulating the displacement and behavior of this small but relevant population of the network [20]. While both discrete and continuous 2D models can recapitulate force transmission anisotropy [22, 24], existing models have difficulty recapitulating the formation of long-range stress cords due to their continuum nature or constraint in the z-axis. The 3D discrete fiber model captures observed long-range force transmission anisotropy [13] and can provide insight into how aligned structures arise in initially unaligned fiber environments, however these long-range fiber strains quickly propagate to the simulation boundaries in relatively few retractions in high sensitivity cells and cells in highly aligned environments. As such, the model’s ability to measure the length-scale of these signals may be limited by the model’s boundary conditions. Further, these results suggest that the model is likely sensitive to a number of network parameters influencing force propagation, such as the average connectivity of nodes within the ECM as well as the ratio between the initial fiber lengths and both the retraction distance and the length-scale of the simulation volume, which were not quantified in this study.

The model’s use of prescribed tractor displacement is likely sufficient for small-scale deformations in the material as each total retraction displaces the ECM 5% the length scale of the volume over the course of 50 steps. However, displacement-controlled tractors may not fully approximate the behavior of stress-controlled tractors nor reflect changes in changes in cell contractility. Experimental data suggest that the contractility behavior [11, 41] are highly dependent ECM density and linear alignment, which both affect ECM stiffness perceived by the cell [36]. While the model simulates changes in cell contractility by increasing tractor density at the polar extents of the cell, contractility could also be modulated by changing the size of the tractors as well as their retraction distance**.** As such, measurements of ECM remodeling may be sensitive to both the size of tractors as well as their contractility, however, this work did not quantify such sensitivities.

The model also interrogates ECM structural anisotropy instead of a perceived stiffness around the cell. While previous work has demonstrated cells exhibit contact guidance behavior even with disrupted integrin binding [30], the model does not currently account for perceived stiffness anisotropy. This perceived stiffness is likely different than bulk measurements measured via macroscale experiments, however, smaller micro-and nano-scale investigations are starting to lend insight into cell-scale ECM perception [42–44]. ECM stiffness may also impact how cells establish tensional homeostasis, which is implicated in remodeling processes [10, 25, 45–47]. As such, a force-dependent tractor model may be able to capture force-dependent phenomena more accurately. Further, the sensitivity expression and the tractor extension and retraction mechanisms are arbitrary and do not account for the considerable literature on the inner workings of the cell, especially contractility [48, 49]. While computational tractability could become an issue, the model could be further developed to incorporate cytomechanical models, like the motor clutch model [50, 51], or additional ECM interrogations, such as local stiffness and steric hinderance [27, 52].

The current modeling framework also lacks an inherent timescale largely due to its approach in tractor retraction. Though it is possible to measure the rates of pseudopod extension and retraction [53], the model simplifies this action by conducting a single large contraction, effectively summing the action of multiple extension and retraction events. Thus the 40 tractors modeled in each retraction event is likely larger than the average number of pseudopod extensions maintained by a cell. These parameters were selected to elicit remodeling behaviors in a computationally tractable manner. The relaxation of the network during after retraction and detachment is also non-physiological and ignores sustained cellular tension. While the current approach can approximates ECM densification patterns and anisotropic strain propagation, time-resolved approaches where tractors probabilistically bind and unbind the ECM may lend additional insight dynamic tension generation, especially if paired with cytomechanical models for actin tension generation [50, 51]. The lack of an apparent timescale may also influence the ECM’s crosslinking behavior. While fiber reorientation and displacement resolve as fast as the retraction process, crosslinking and entanglement may occur on a longer timescale. Further approaches that break up the mass retraction process into individual time-resolved events and a probabilistic model for fiber-fiber bonding may be able to account for these in future models.

Finally, this work presents a customizable framework that can increase its functionality by leveraging existing software packages and LAMMPS’ built-in functions. While the presented work used Voronoi and Delaunay tessellations, additional network topographies could be simulated [25]. Further, while the networks were simulated as networks of homogenous fibers, fiber-specific parameters such as diameter and stiffness could be altered upon network generation by defining different molecule types and different rules of interaction. These interaction rules could be further extended to prevent fibers of a certain type from binding to other fibers or to change universal crosslinking rules. Additional fiber properties like bending stiffness can be added to fiber segment definitions, conferring additional resistance to bending within simulated networks. Further, fiber segments can be repeatedly updated between retraction steps to change to fiber spring constants, resting distances, and Lennard-Jones length constants to simulate the fiber stiffening or thickening dependent on the stress state of fibers or fiber segments. However, the creation of many different types of fibers and additional rules for interaction would necessarily add to the computational complexity and memory demands of such simulations. Finally, the rules governing fiber-fiber interactions can be further extended. While fiber-fiber crosslinks formed deterministically in the presented results, fiber crosslinking can also be probabilistically determined, promoting bond creation between fibers that contact each other longer than transient interactions. Bond breakage can also be handled by existing functions in LAMMPS, enabling stress-dependent bond breakage within the fiber network. Bond breakage was not implemented in these experiments due to the nature of the static cell and the potential detachment of the network nearest the cell from the rest of the ECM. The addition of such parameters would increase the computational cost of simulations, but LAMMPS’ parallelizable structures and GPU implementations may be leveraged to increase computational tractability.

Despite these shortcomings, the model presents an open-source and expandable framework that synthesizes elements of cell ECM-feedback mechanisms and ECM plasticity to provide insight into ECM heterogeneity at the fiber level. The current implementation of the model can capture experimentally observed cell and ECM remodeling trends and the model’s modular nature will enable further development in this framework with adjustable methods for cell ECM sensing, cell sensitivity, and tractor manipulation. Finally, the model’s implementation in an open-source molecular dynamics framework lends itself to parallelization and cluster-based computing.

## Electronic supplementary material

Below is the link to the electronic supplementary material.

Supplementary file1 (DOCX 2764 kb)

## Data Availability

The scripts necessary to run the model are publicly available on github: (https://github.umn.edu/Barocas-Research-Group/TRACTORS). Data is available upon request.

## Appendices

Equations

$$\upvarphi =\frac{\sum_{f}{l}_{f}\pi {r}^{2}}{V}$$

$${E}_{bond}\left(r\right)=K{\left(r-{r}_{0}\right)}^{2}$$

$${E}_{pair}(r)=\left\{\begin{array}{c}4{\varepsilon}_{f}\left[{\left(\frac{{\sigma}_{f}}{r}\right)}^{12}-{\left(\frac{{\sigma}_{f}}{r}\right)}^{6}\right], r<{r}_{cf}\\ 0, r\ge {r}_{cf}\end{array}\right.$$

$$\begin{array}{cc}E({r}_{1},{r}_{2},\dots ,{r}_{N})=& \sum_{i,j}{E}_{pair}({r}_{i},{r}_{j})+\sum_{ij}{E}_{bond}({r}_{i},{r}_{j})\\ & \end{array}$$

$$\boldsymbol{\rm X}=\frac{{{\boldsymbol{\Omega}}}^{s}}{trace\left({{\boldsymbol{\Omega}}}^{s}\right)}, where {{\boldsymbol{\Omega}}}^{s}\equiv {\boldsymbol{V}}\left[\begin{array}{ccc}{\Omega}_{1}^{s}& 0& 0\\ 0& {\Omega}_{2}^{s}& 0\\ 0& 0& {\Omega}_{3}^{s}\end{array}\right] {{\boldsymbol{V}}}^{{\boldsymbol{T}}}$$

$$\boldsymbol{\Omega }=\frac{1}{\sum_{m}{L}_{m}}\sum_{m}{L}_{m}{{\boldsymbol{n}}}_{{\boldsymbol{m}}}\otimes{{\boldsymbol{n}}}_{{\boldsymbol{m}}}$$

## Declarations

### Citation Diversity

Recent work has identified a bias in citation underrepresenting underciting women and other minority scholars. We recognize this bias and reference papers with fair gender and racial author inclusion.
