# Supplementary material for: Cell-Extracellular Matrix Feedback Results in Spontaneous Cell Polarization and Heterogeneous Remodeling in 3D Isotropic and Aligned Discrete-Fiber Models of Cell-Mediated Remodeling
Source: Cell Mol Bioeng. 2026 Jun 20;19(3):407–26. doi: 10.1007/s12195-026-00922-0 (PMC13365295; doi:10.1007/s12195-026-00922-0)
Supplement: Supplementary file 1 — Supplementary file1 (DOCX 2764 kb) [file 12195_2026_922_MOESM1_ESM.docx]

# Supplementary Figures


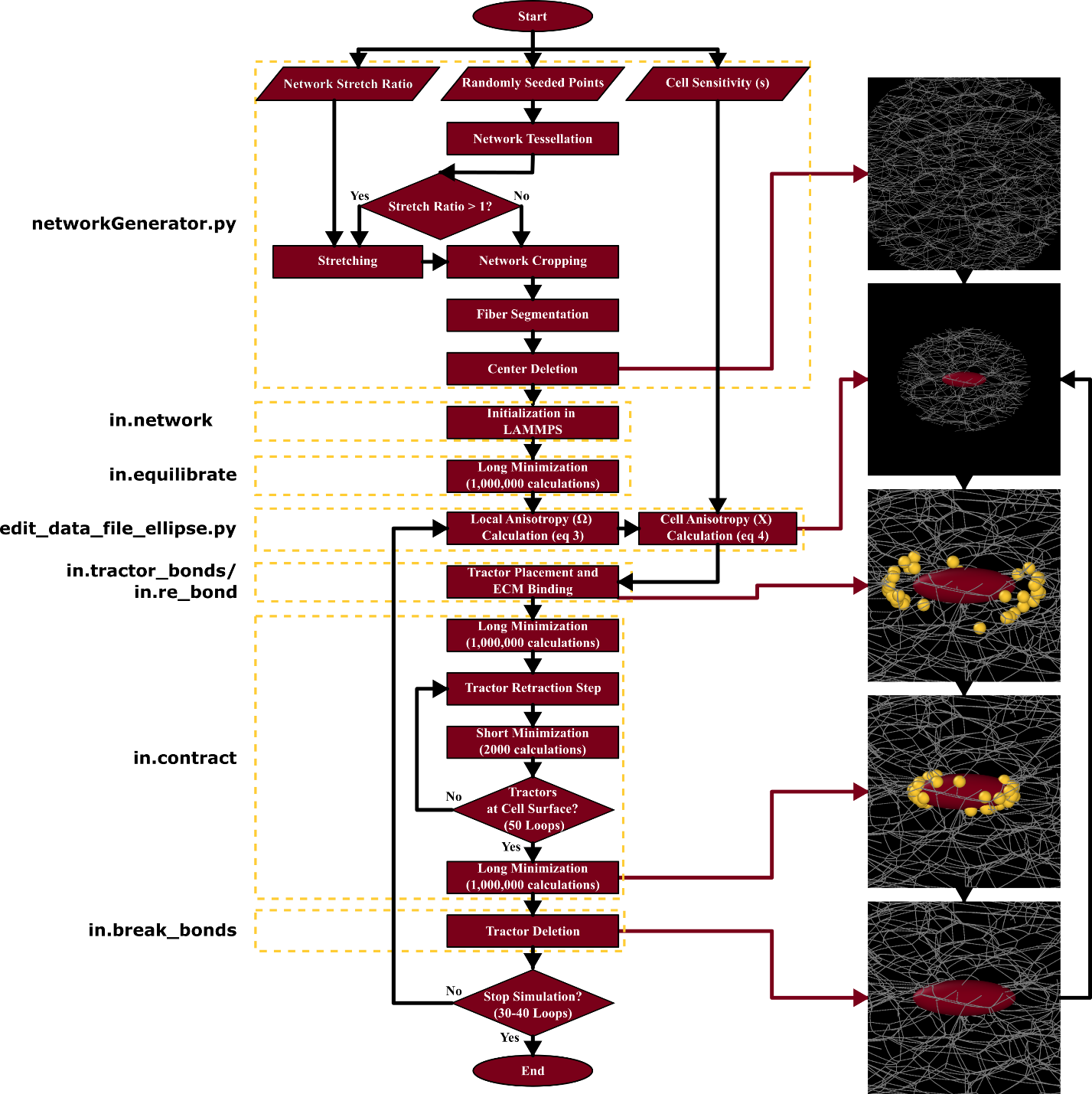


**Figure S1**: A flowchart of the simulation protocol with names of scripts (left) and snapshots of the simulation state (right).


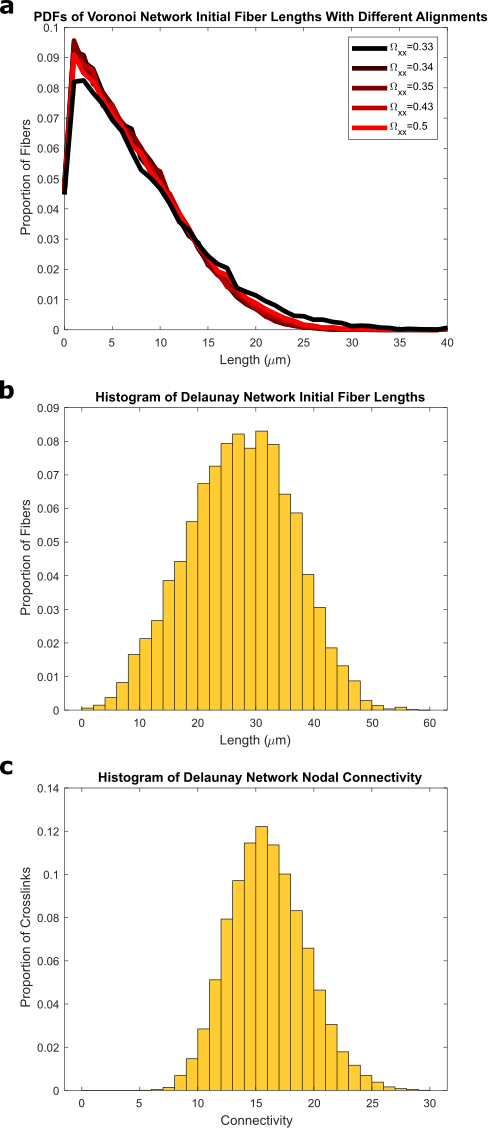


**Figure S2:** (a) The probability distribution function of fiber lengths in Voronoi networks with increasing pre-alignment (black to red). N=10 for each alignment. (b) The distribution of fiber lengths among 10 Delaunay tessellations used in the simulation. (c) The distribution of node connectivity among 10 Delaunay tessellations.
